# Dry Eye Disease and Psychiatric Disorders: Neuroimmune Mechanisms and Therapeutic Perspectives

**DOI:** 10.3390/ijms262110699

**Published:** 2025-11-03

**Authors:** Snježana Kaštelan, Lea Kozina, Zora Tomić, Ivana Bakija, Tomislav Matejić, Domagoj Vidović

**Affiliations:** 1 Department of Ophthalmology, Clinical Hospital Dubrava, School of Medicine, University of Zagreb, 10000 Zagreb, Croatia; 2 Department of Sleep Disorders, University Psychiatric Hospital Vrapče, 10000 Zagreb, Croatia; 3 Health Centre of the Croatian Department of Internal Affairs, 10000 Zagreb, Croatia; 4 Department of Integrative Psychiatry, University Psychiatric Hospital “Sveti Ivan”, 10090 Zagreb, Croatia; 5 Surgery Clinic, Clinical Hospital Sveti Duh, 10000 Zagreb, Croatia

**Keywords:** dry eye disease, psychiatric disorders, sleep disturbances, neuroimmune pathways, hypothalamic–pituitary–adrenal axis dysregulation, brain-derived neurotrophic factor, oxidative stress, tear biomarkers, psychotropic medications, integrated care

## Abstract

Dry eye disease (DED) is a highly prevalent multifactorial disorder of the ocular surface that extends beyond local tear film pathology to involve systemic immune, neuroendocrine, and neurosensory mechanisms. Increasing evidence reveals a strong and bidirectional association between DED and psychiatric disorders, particularly depression, anxiety, post-traumatic stress disorder (PTSD), and sleep disturbances. This review synthesises the current knowledge on shared molecular, neuroimmune, and neuropathic pathways that underlie this comorbidity. Key mechanisms include hypothalamic–pituitary–adrenal (HPA) axis dysregulation, systemic and ocular inflammation, oxidative stress, mitochondrial dysfunction, and impaired neurotrophic signaling, especially reduced brain-derived neurotrophic factor (BDNF). Dysregulation of monoaminergic neurotransmitters such as serotonin and norepinephrine not only contributes to mood disturbances but also alters tear secretion and corneal pain perception. Corneal nerve changes and trigeminal–limbic sensitisation further reinforce the overlap between neuropathic ocular pain and affective dysregulation. Psychotropic medications, while essential for psychiatric care, may exacerbate ocular surface dysfunction through anticholinergic effects, altered neurotransmission, and tear film instability, highlighting the iatrogenic dimension of this interface. Conversely, tear-based biomarkers, including cytokines, serotonin, and BDNF, offer promising translational tools for patient stratification, diagnosis, and treatment monitoring across ocular and psychiatric domains. Recognising DED as part of a systemic, biopsychosocial continuum is critical for effective management. Multidisciplinary strategies that integrate ophthalmologic and psychiatric care, alongside novel therapies targeting shared molecular pathways, provide a framework for improving outcomes. Future research should prioritise longitudinal studies, biomarker validation, and personalised interventions to address this complex comorbidity.

## 1. Introduction

Dry eye disease (DED) is a chronic, multifactorial disorder of the ocular surface characterised by loss of tear film homeostasis, ocular discomfort, visual disturbance, and inflammation. Affecting up to 50% of individuals in certain populations, especially older adults and postmenopausal women, DED has become a significant global public health concern. While traditionally considered a local ocular surface pathology, a growing body of evidence highlights its systemic dimensions and interrelationships with other chronic conditions, particularly psychiatric disorders [1,2,3].

Over the past decade, studies have shown a strong comorbidity between DED and psychiatric conditions, especially depression, anxiety, post-traumatic stress disorder (PTSD), and sleep disturbances. Epidemiological evidence confirms that psychiatric symptoms are significantly more common in DED patients than in the general population [4,5,6,7,8,9,10,11,12]. These psychiatric comorbidities may exacerbate symptom perception, especially ocular pain and dryness, while chronic ocular symptoms themselves can contribute to psychological distress, creating a bidirectional feedback loop [13,14,15,16,17,18,19,20,21,22]. Discrepancies between subjective symptoms and objective signs in DED are common and are frequently attributed to somatisation, alexithymia, and central sensitisation mechanisms [12,13,14,15,18,23].

Epidemiological studies reveal that up to 54% of patients with DED suffer from depression, and 64% report anxiety symptoms [5,6]. Sleep disturbance is even more prevalent, affecting more than 80% of DED patients [5,24,25]. These psychiatric comorbidities not only exacerbate the subjective experience of ocular symptoms, especially pain and dryness, but also contribute to disease chronification, diminished treatment response, and impaired quality of life. Conversely, chronic ocular discomfort, blurred vision, and visual fatigue serve as persistent stressors that may induce or worsen psychiatric symptoms, creating a vicious cycle of somatic and psychological burden [5,6,7,9,11,14,26].

The symptom–sign discordance in DED highlights its bidirectional link with psychiatric disorders [12,13,14,15]. Molecular and neurobiological studies suggest common pathways that may explain this overlap [5,6,7,23]. Both DED and psychiatric disorders are linked to chronic systemic inflammation, hypothalamic–pituitary–adrenal (HPA) axis dysregulation, oxidative stress, mitochondrial dysfunction, and reduced levels of neurotrophic factors like brain-derived neurotrophic factor (BDNF). Dysregulated neurotransmitter systems, particularly serotonin and norepinephrine, play vital roles in mood regulation, ocular surface stability, tear secretion, and pain perception [26,27,28]. Tear serotonin levels have been correlated with DED severity and ocular pain, suggesting potential as a biomarker [29].

From a neuropathic perspective, in vivo confocal microscopy studies reveal structural changes in the corneal nerve plexus of DED patients, including decreased nerve density, increased tortuosity, and formation of microneuroma. These alterations are associated with symptoms of hyperalgesia and allodynia, and mirror the central sensitisation phenomena seen in chronic pain syndromes and mood disorders [13,30,31,32]. Additionally, several neuropeptides involved in pain signalling and emotional regulation, such as substance P, Calcitonin Gene-Related Peptide (CGRP), and Transient Receptor Vanilloid 1 (TRPV1), are dysregulated in DED [33,34,35].

Adding further complexity, psychotropic medications commonly prescribed for depression and anxiety, including selective serotonin reuptake inhibitors (SSRIs) and selective serotonin and norepinephrine reuptake inhibitors (SNRIs), tricyclic antidepressants (TCAs), and antipsychotics, may impair ocular surface function through anticholinergic effects, tear reflex suppression, or immune response modulation. This creates an important iatrogenic aspect to the DED-psychiatry interface, especially among elderly patients and those on polypharmacy [6,7,10,11,13,20,23].

Despite the accumulating evidence, the connection between DED and psychiatric disorders remains under-recognised in clinical practice [6,16]. Ophthalmologists often overlook psychiatric contributors to symptom severity, while mental health professionals may misattribute ocular symptoms to medication side effects. A biopsychosocial approach and interdisciplinary collaboration are essential for optimising diagnosis and treatment in this patient population [23,36,37,38,39].

This review synthesises the current knowledge on the shared molecular, neuroimmune, and neuropathic mechanisms underlying the connection between DED and psychiatric disorders. It presents emerging insights into the involvement of BDNF, oxidative stress, HPA axis dysregulation, and serotonin signalling as potential links between ocular and mental health. By integrating molecular findings with clinical observations, this review proposes a biopsychosocial framework to support more effective diagnosis, biomarker identification, and multidisciplinary treatment strategies. Emphasising a translational perspective, this review aims to facilitate the integration of ocular and psychiatric care, ultimately improving patient outcomes.

### Data Collection

A comprehensive narrative literature review was conducted to provide an integrative overview of the bidirectional relationship between DED and psychiatric disorders. The specific objectives were to summarise the epidemiological associations, shared aetiological and pathophysiological mechanisms, overlapping symptomatology, clinical implications, emerging therapeutic strategies, and future research perspectives. A systematic literature search was conducted using the PubMed, MEDLINE, and Google Scholar databases, encompassing all publications available up to June 2025. The search strategy incorporated a combination of the following keywords: “dry eye disease”, “dry eye”, “ocular surface”, “tear”, “psych*”, “psychiatry*”, “psychiatric disorders”, “psychiatric symptoms”, “psychological”, “depress*”, “depression”, “anxiety”, “sleep”, “stress”, “mechanism”, “neuroimmune”, “neuroimmune mechanisms”, “molecular”, “pain”, “medications”, “psychotropic”, “drugs”, “biomarkers”, “therapy”, “treatment”, and “interventions”. The search was limited to studies published in English. After the initial identification of relevant publications, duplicates were removed, and the titles and abstracts were screened to assess eligibility. Full-text articles that met the inclusion criteria were subsequently reviewed in detail. The reference lists of selected studies were manually examined (snowballing method) to identify additional relevant articles not captured in the initial database search. All types of peer-reviewed scientific publications written in English were considered, including original preclinical and clinical research, systematic reviews, and meta-analyses. To ensure inclusion of the most up-to-date evidence, particular emphasis was placed on studies published within the past five years. Articles without English translations, those not aligned with this review’s objectives, and those failing to meet higher methodological or publication standards were excluded. No quantitative data synthesis was performed, as the aim was to provide a comprehensive narrative discussion on the topic. This review is therefore designed as a narrative synthesis aimed at integrating mechanistic and clinical evidence rather than performing quantitative meta-analysis.

## 2. Epidemiological and Psychosomatic Links Between Dry Eye Disease and Psychiatric Disorders

DED is closely linked with psychiatric disorders, influencing symptom severity, quality of life, and treatment outcomes [5,6,7,8,9,11,12,13,14,15,16,20,23,27]. Understanding these interactions is essential for comprehensive patient care and the development of personalised therapeutic strategies, which is key to holistic patient management.

### 2.1. Psychiatric Comorbidities and Symptom–Sign Discordance

Patients with DED commonly exhibit psychiatric symptoms, with significantly higher rates of depression, anxiety, and sleep disturbances compared to the general population. Meta-analyses estimate that 40–54% of DED patients experience depression, while 39–64% report anxiety symptoms [5,6]. Conversely, nearly one-third of individuals with depression and anxiety are diagnosed with DED, and 22% report DED-like symptoms without a formal diagnosis [6]. A large meta-analysis of approximately three million participants showed that DED patients have a threefold higher risk of both depression and anxiety than controls [7].

Sleep disturbances are prevalent in DED, affecting up to 84% of patients [5]. These include insomnia, sleep fragmentation, and obstructive sleep apnea [8], with women particularly affected [4,9]. Among ocular conditions, DED is linked to the worst sleep outcomes, with over 42% of patients reporting disturbed sleep [9]. Depression is especially pronounced in subgroups such as patients with Sjögren’s syndrome [7].

Emerging data also point to a potential association between PTSD and DED. In a U.S. Veterans Affairs cohort, women with PTSD had a 2.4-fold increased risk of DED compared to men [10]. Additionally, obsessive compulsive disorder (OCD) has been associated with heightened DED symptomatology [11]. These findings underscore the multidimensional burden of DED and the need for integrated psychiatric screening.

A hallmark feature of DED is the poor and inconsistent correlation between subjective symptom severity and objective clinical signs [12,13]. This disconnection resembles patterns seen in other chronic diseases like rheumatoid arthritis and glaucoma, where patients may exhibit marked signs without symptoms, delaying diagnosis and treatment [14,15]. Approximately 42% of patients show this discrepancy, either reporting significant symptoms without objective evidence or presenting with clinical signs in the absence of symptoms [15].

This disconnection is particularly prominent in patients with anxiety or depression. Psychiatric symptoms correlate strongly with subjective complaints but not with tear break-up time (TBUT), Schirmer test results, or fluorescein staining. This may reflect altered perception due to disrupted neurochemical signalling in mood disorders, which amplifies perception of somatic symptoms [16]. Patients often report higher depressive symptoms on self-rating scales, yet structured psychiatric interviews reveal fewer actual diagnoses, suggesting emotional hypersensitivity or somatisation [16,39].

A similar dissociation is seen with sleep disturbances. Tools like the Insomnia Severity Index (ISI) and the Pittsburgh Sleep Quality Index (PSQI) correlate with subjective symptoms but not with objective ocular surface metrics [17]. Pain perception appears central to this disconnection. Ong et al. identified pain as a key factor underlying the mismatch between subjective symptoms and objective signs in DED, particularly in patients with arthritis, extraocular pain, or central sensitisation syndromes [13]. Other contributing factors include female sex, likely due to hormonal influences, and sensitivity to low humidity [15]. This discrepancy may, at least in part, be attributed to the inherent limitations in sensitivity, specificity, and reproducibility of current diagnostic tests. Many objective tests show poor reproducibility and low intercorrelation, and are most effective only in advanced DED. Tear osmolarity remains the most consistent measure across all disease severities [12]. Symptom variability during the day, often worse in the evening, further complicates assessment. Patients with primary Sjögren’s syndrome may report fewer symptoms despite severe objective findings, possibly due to altered pain signalling involving TRPV1 and substance P [18].

Older patients may underreport symptoms due to age-related reductions in corneal sensitivity and a stoic personality profile [13,19]. Notably, individuals with the greatest symptom–sign discrepancies tend to have lower corneal nerve density and worse ocular-related quality of life [16,20,23]. As in other chronic illnesses, this discrepancy may impair treatment response [21]. Notably, up to 18% of pilots demonstrate objective signs of DED without symptoms, raising safety concerns and highlighting the need for improved diagnostic protocols [19].

### 2.2. Psychological Factors in Symptom Amplification

In addition to biological mechanisms, psychological factors such as somatisation significantly influence symptom perception in DED [16,22]. Somatisation, defined as the experience of physical symptoms without objective findings, affects up to 80% of individuals with depression and frequently co-occurs with anxiety and depressive symptoms [5,24,25]. DED patients with pronounced symptoms but minimal signs often exhibit high levels of somatisation and alexithymia, suggesting that emotional dysregulation may manifest as ocular discomfort [24,26].

#### 2.2.1. Somatisation and Central Pain Sensitisation

Somatisation plays a key role in pain amplification and treatment resistance, contributing to the subjective burden of DED, particularly ocular pain, which strongly correlates with anxiety, depression, and sleep disturbances [7,9]. Health anxiety has also been identified as a mediator linking psychiatric symptoms with dry eye complaints [36]. These findings underscore the importance of assessing psychological dimensions during DED evaluations.

While somatic symptoms are part of depression’s diagnostic criteria, they should not be equated with somatisation [37]. In some cases, DED may be a somatic expression of depression, given overlapping mechanisms such as pain sensitisation [37,38]. However, although pain sensitisation and somatisation are related, they remain distinct entities [40]. It is therefore plausible that somatisation amplifies rather than causes DED symptoms [36].

#### 2.2.2. Personality Traits and Emotional Vulnerability

Several personality traits and psychological factors, such as neuroticism, low conscientiousness, emotional dysregulation, and reduced subjective well-being, have been associated with increased DED symptom severity [5,16,27,41]. Anxiety sensitivity is another contributor, particularly under emotional stress [42].

Lifestyle factors such as excessive screen time contribute to both DED and poor sleep quality [17,28,43], while poor sleep exacerbates stress, anxiety, and DED symptoms [17,20]. Typical DED symptoms, such as ocular pain, foreign body sensation, and blurred vision, negatively affect quality of life and may precipitate depressive symptoms [16]. Frequent medical visits, low treatment satisfaction, and the financial burden of care further reinforce emotional distress [16,22].

Evidence supports a strong, bidirectional association between DED and psychiatric symptoms [14,44,45,46]. Shared risk factors include female sex, menopause, ageing, and an elevated omega-6:omega-3 ratio [5,8,20,46]. Patients with moderate-to-severe DED often report a higher psychiatric burden, particularly in postmenopausal women with Sjögren’s syndrome [5,7,8,38].

#### 2.2.3. Sleep Disturbances and Lifestyle Risk Factors

Sleep disturbances are both a consequence and a driver of psychiatric and ocular symptoms. Individuals sleeping five or fewer hours per night report significantly worse DED symptoms [47]. Among DED symptoms, ocular pain shows the strongest association with depression, anxiety, and sleep disorders [9], while blurred vision is more closely related to depression than anxiety [14,48]. In severe cases, sleep quality appears to be the strongest predictor of symptom burden. Meibomian gland dysfunction and ocular surface inflammation are both associated with poor sleep [17].

#### 2.2.4. Implications for Clinical Management and Research

An observational study on DED treatment over 3–6 months showed reduced therapeutic efficacy in patients with comorbid anxiety or depression. Patients without psychiatric comorbidity experienced greater symptom relief, and while DED therapy somewhat improved depressive symptoms, it had no significant effect on anxiety [14]. These findings support routine screening for dry eye symptoms in individuals with psychiatric conditions, with early referral to ophthalmology when needed [8].

Despite growing interest, longitudinal studies examining causal links between psychiatric disorders and DED remain limited [6,16]. Many reports are based on small, cross-sectional samples. They should therefore be interpreted cautiously, as high prevalence estimates may reflect selection bias or subjective symptom reporting rather than true population effects [7,12]. Psychiatric–ocular research is particularly prone to methodological variability, including inconsistent diagnostic criteria, heterogeneous assessment tools, small sample size and the lack of a control group in many studies [7,8,10,11,12,26]. Selection bias and reliance on self-reported symptoms further challenge the validity of observed associations [7,45]. Moreover, most studies do not clearly distinguish between primary DED and secondary forms related to psychopharmacologic treatment, which may confound interpretation of pathophysiological mechanisms and therapeutic outcomes. Geographic, sociocultural, and epigenetic factors also influence disease expression and likely contribute to the heterogeneity reported in meta-analyses [14]. Sociocultural and epigenetic influences may further shape the clinical expression and perception of DED–psychiatric comorbidity [5,6,14,16,20,23]. Studies from Asian cohorts often show higher DED prevalence, especially among women, reflecting differences in lifestyle, diet, and cultural norms affecting symptom reporting and healthcare-seeking behaviour. Further, data from U.S. Veterans highlight occupational stress, trauma exposure, and psychotropic medication use as major contributing factors [4,5,6,7,8,9,10,11,13,20,23]. At the molecular level, stress, sleep deprivation, and inflammation can trigger epigenetic modifications in genes via regulation of the HPA axis and neurotrophic signaling such as *BDNF* and *NR3C1*, leading to population-specific vulnerability patterns [14,16,17,20,23]. Integrating these sociocultural and biological determinants into future research frameworks will improve comparability between studies and strengthen causal inference.

Future research should therefore employ standardised diagnostic protocols for both ocular and psychiatric evaluation, include well-defined control groups, and use validated, clinician-administered instruments [16]. Large, multicentre, longitudinal studies are needed to minimise cultural and regional variability and strengthen causal inference. Given the complex interaction of ocular, psychiatric, and somatic factors, future work should also utilise advanced statistical approaches, such as cluster or latent class analyses, to identify high-risk patient phenotypes and guide the development of personalised and integrated therapeutic strategies [49].

## 3. Shared Molecular and Neuroimmune Mechanisms

Emerging evidence highlights a significant overlap in the molecular and neuroimmune pathways underlying DED and psychiatric disorders. Table 1 provides a summary of the principal shared molecular and neuroimmune factors implicated in DED and psychiatric conditions [5,16,20,28,29,50,51,52,53,54,55,56,57,58,59,60,61,62,63,64,65,66,67,68,69,70,71,72,73,74,75,76,77,78,79,80,81,82,83,84,85,86,87,88,89,90,91,92,93,94,95,96,97,98,99,100,101,102,103,104,105,106,107,108,109,110,111,112,113,114,115,116,117,118,119,120,121,122,123,124,125].

### 3.1. HPA Axis Dysregulation and Stress-Immune Interactions

Chronic stress triggers prolonged activation of the HPA axis, elevating levels of corticotropin-releasing hormone (CRH) and cortisol, central mediators in the pathophysiology of depression and anxiety [51,52]. In depressive states, glucocorticoid receptor resistance impairs negative feedback regulation, perpetuating HPA axis hyperactivity and increasing cortisol secretion [51,52,53]. Excess cortisol is linked to hippocampal and prefrontal cortical atrophy, reduced neurogenesis, and synaptic remodelling deficits that underlie mood disturbances [53].

HPA axis dysregulation promotes neuroinflammation, which both drives and results from depressive pathology, creating a vicious cycle of stress and immune activation [51,53]. Disruptions in the gut microbiota, frequently observed in psychiatric disorders, may further stimulate the HPA axis through immune signalling molecules, microbial metabolites, and gut-derived peptides, reflecting the complexity of the gut–brain–immune axis [53].

Elevated cortisol and C-reactive protein (CRP) levels are more strongly associated with somatic depressive symptoms, such as sleep disturbance, than with affective or cognitive dimensions [54]. Similar neuroendocrine alterations are observed in PTSD, anxiety, and other stress-related conditions [5,8,14,51,55], with direct relevance to DED. Individuals in high-stress occupations show an increased prevalence of DED, likely mediated through chronic HPA axis activation [56]. Patients with Sjögren’s syndrome display elevated salivary cortisol and greater psychiatric symptom burden than healthy controls [57]. Moreover, tear cortisol levels correlate positively with anxiety and DED symptom severity, implicating stress-induced endocrine changes in enhanced pain sensitivity, a key feature of DED [56,58].

Sleep disturbances intensify these effects by altering diurnal cortisol rhythms, elevating evening levels, and increasing the cortisol awakening response, thereby exacerbating both insomnia and ocular surface dysfunction [5,59]. Central CRH-mediated arousal mechanisms may further reinforce this maladaptive cycle [52]. These findings underscore the potential of tear cortisol as a non-invasive biomarker connecting psychiatric symptomatology and DED pathophysiology [58].

### 3.2. Monoaminergic Imbalance and Pain Modulation

Altered monoaminergic signalling, particularly involving serotonin, norepinephrine, and dopamine, plays a central role in the pathophysiology of depression and contributes to somatic symptoms, including chronic pain [60,63]. Reduced synaptic levels of these neurotransmitters in depression are associated with low mood, cognitive impairment, fatigue, and disturbed sleep. Antidepressants exert their effects by increasing monoamine availability through reuptake inhibition or enhanced neurotransmitter release [61].

Depression and pain share overlapping neurobiological mechanisms, with chronic pain often leading to depressive symptoms and vice versa [62,64,119]. Individuals with depression exhibit increased pain sensitivity, largely due to central sensitisation, a maladaptive amplification of nociceptive signals in the central nervous system [62,120]. This process is exacerbated by comorbid anxiety and sleep disturbances. Monoaminergic systems modulate both ascending and descending pain pathways, playing a critical role in regulating synaptic plasticity, including long-term potentiation and depression, which underlie central sensitisation [62,65,66].

The descending pain modulatory system originates in brainstem nuclei, including serotonergic projections from the raphe nuclei, noradrenergic fibres from the locus coeruleus, and dopaminergic inputs from the ventral tegmental area. These pathways regulate nociceptive transmission at the spinal dorsal horn [62,64,67]. Ascending monoaminergic projections convey pain-related signals through the thalamus to cortical regions involved in sensory and emotional processing, such as the insula, anterior cingulate cortex, prefrontal cortex, amygdala, and hippocampus [64,68].

Pain perception is regulated by central brain regions, including the cingulate cortex, thalamus, insula, prefrontal cortex, amygdala, and cerebellum, where monoaminergic neurotransmission plays a crucial role. Serotonin, dopamine, and norepinephrine modulate both pathways. Descending from the raphe nuclei, locus coeruleus, and ventral tegmental area to the spinal cord, and ascending that relay pain signals via the thalamus to the somatosensory cortex [62,64]. Emotional processing centres, such as the amygdala, prefrontal cortex and insula, further influence pain perception via monoaminergic inputs, underscoring the anatomical and functional overlap between pain and emotion in psychiatric disorders and DED [62,68].

Pain is a common symptom in DED and may, in some patients, persist as chronic ocular pain. Monoaminergic dysregulation is implicated in this process. Elevated tear serotonin has been observed in patients with low tear secretion and prominent symptoms. Others with preserved tear function report photophobia and wind sensitivity, suggesting neuropathic pain and central sensitisation [29]. These symptoms may occur independently of ocular surface damage. Moreover, DED patients with comorbid conditions such as depression or PTSD often report more severe pain [69], supporting a role for central monoaminergic mechanisms in amplifying DED-related discomfort.

### 3.3. Proinflammatory Cytokines in Ocular and Mental Health

Chronic stress, a major contributor to psychiatric disorders, induces low-grade systemic inflammation characterised by elevated proinflammatory cytokines. These cytokines disrupt neurotransmitter signalling, impair glucocorticoid feedback, and contribute to HPA axis dysregulation, fostering depressive states [50,51,60]. Patients with depression often exhibit increased levels of interleukin-6 (IL-6), IL-1β, IL-10, tumour necrosis factor-α (TNF-α), CRP, and transforming growth factor-β (TGF-β), many of which are produced in response to cellular stress through inflammasome activation [51,60]. Monocyte-driven inflammation further disrupts HPA axis homeostasis, perpetuating cortisol elevation and immune dysregulation. Chronic stress and early-life inflammatory insults are crucial triggers for this proinflammatory state [70].

Neuroinflammation impairs neurogenesis and plasticity through the IL-1β-induced activation of the kynurenine pathway, TNF-α-mediated glutamate excitotoxicity via microglial activation, increased blood–brain barrier permeability, and suppression of serotonin synthesis [51,53,70]. Persistent inflammation also induces epigenetic changes that sustain the proinflammatory environment, worsening depressive symptoms [39]. Elevated cytokines such as IL-10, IL-1β, and TNF-α are linked to common comorbidities of depression, including hypertension and diabetes, and may serve as potential markers of disease severity and treatment response [51,60].

Monoamines regulate peripheral immune responses: norepinephrine acts via β2-adrenoceptors to suppress inflammation, serotonin modulates T cell function through 5-HT receptors, and dopamine influences various immune cells [71]. Pharmacological evidence indicates that antidepressants, including SSRIs, SNRIs, monoamine oxidase inhibitors (MAOIs), and TCAs, exhibit anti-inflammatory effects by reducing proinflammatory cytokines and quinolinic acid while increasing anti-inflammatory cytokines and kynurenic acid [60,70]. These findings suggest that targeted immunotherapies and anti-inflammatory interventions may have potential in alleviating depressive symptoms [60]. DED similarly involves inflammation by increasing cellular inflammation in the conjunctiva and cornea as well as elevated tear levels of IL-6 and IL-17, particularly in Sjögren’s syndrome [16,72]. High IL-17 and TNF-α levels correlate with DED severity and progression [16]. Studies reveal that depressed patients with DED have higher tear concentrations of these cytokines compared to controls [16,72]. However, the current evidence does not consistently support inflammatory mediators as direct mechanistic links between depression and DED severity, underscoring the need for further investigation [38].

### 3.4. Oxidative Stress, Mitochondrial Dysfunction and Neurotrophic Signalling

Reduced BDNF signalling, oxidative stress, and mitochondrial dysfunction converge to disrupt neuronal and ocular surface homeostasis. Impaired BDNF weakens neurotrophic support, excessive ROS damages cells and suppresses BDNF activity, and mitochondrial deficits amplify energy failure and oxidative injury. Together, these interconnected pathways provide a mechanistic link between ocular surface pathology and mood disorders, highlighting potential targets for therapeutic intervention [74,75,76,77,78,79,80,85,86,87,88,89,90,91,92,93,94,95,96,97,98,99,100,101,102,103].

#### 3.4.1. BDNF Signaling at the Ocular–Psychiatric Interface

BDNF is a key neurotrophin regulating synaptic plasticity, neuronal survival, and neuroimmune signalling. Reduced peripheral BDNF levels are observed in various chronic psychiatric and neurological disorders, correlating with symptom severity and duration [73]. This reduction often involves impaired processing of proBDNF to mature BDNF, further compromising neurotrophic signalling [74]. In the eye, BDNF is produced by corneal sensory neurons and epithelial cells, interacting with highly expressed tyrosine kinase (Trk) receptors [75,121]. Preclinical studies suggest that BDNF contributes to tear film homeostasis and ocular surface integrity [76].

Polymorphisms in the BDNF receptor gene, such as Val66Met, have been linked to ophthalmological symptoms, including DED and may mediate the interaction between depression and DED [20,77,78]. Elevated circulating BDNF levels have been reported in patients with Sjögren’s syndrome, possibly reflecting a compensatory response to chronic ocular inflammation [78]. Chronic DED induces local corneal inflammation and increased BDNF expression in the trigeminal ganglion, which may enhance ocular pain perception [26,27,28,78]. Moreover, stress-induced reduction in tear secretion may be mediated via altered BDNF signalling pathways, linking psychological stress to worsening DED symptoms [76].

Tear BDNF has emerged as a promising biomarker for ocular diseases, given its correlation with disease severity and symptomatology [121,122]. A preclinical study on mice demonstrates that activation of Trk receptors by selective agonists alleviates DED symptoms through modulation of the NF-κB pathway and suppression of inflammation, a mechanism analogous to the effects of corticosteroids on Trk signalling [75]. BDNF may also serve as a link between neurodegenerative and inflammatory processes in ocular diseases, as shown in uveoretinitis mouse models [123], which may partly explain the elevated prevalence of DED in Parkinson’s disease, where BDNF signalling is impaired [124]. Furthermore, recent clinical findings highlight tear BDNF as a predictor of mental health and sleep quality in patients with Sjögren’s syndrome-associated DED, emphasising the complex interplay between ocular and neuropsychiatric pathways [79].

The role of BDNF in depression pathophysiology is well established. Reduced BDNF levels are consistently reported in brain regions critical for emotion regulation, such as the hippocampus, limbic system, and frontal cortex [53,74]. Chronic stress impairs BDNF expression and BDNF–TrkB signalling, leading to synaptic dysfunction and neuronal atrophy [51,74,80]. Elevated cortisol levels from HPA axis dysregulation further suppress BDNF, while gut microbiota imbalances commonly observed in depression also diminish BDNF activity [53]. Consequently, therapeutic strategies aimed at restoring BDNF signalling are being explored as potential interventions for depression and associated neuropsychiatric disorders [74].

#### 3.4.2. Oxidative Stress and Redox Imbalance

Neurons’ high oxygen demand makes them particularly susceptible to oxidative stress and its byproducts, such as 8-hydroxy-2′-deoxyguanosine and malondialdehyde [81,82]. Oxidative stress contributes to neuronal injury in depression directly and through HPA axis dysregulation, particularly hypercortisolemia-induced hippocampal atrophy [39]. Inflammation and oxidative stress act synergistically [53,85], as pro-inflammatory cytokines divert tryptophan metabolism toward quinolinic acid, thereby amplifying oxidative damage and suppressing BDNF expression [53]. Oxidative damage also correlates with depressive symptom severity [82,83,84].

BDNF counteracts oxidative stress via nuclear factor erythroid 2-related factor 2 (Nrf2) activation, inducing antioxidant enzymes such as superoxide dismutase, glutathione peroxidase, and NAD(P)H:quinone dehydrogenase 1 [81,125]. Dysregulation of methyl-CpG-binding protein 2 (MeCP2) disrupts redox homeostasis and reduces hippocampal BDNF, linking oxidative imbalance to neurotrophic dysfunction [86,87].

Certain antidepressants, including SSRIs, could mitigate oxidative injury by lowering malondialdehyde levels, and antioxidant-rich diets may provide adjunctive benefit. Reactive nitrogen species intensify oxidative damage through peroxynitrite formation, whereas paroxetine exhibits protective effects [28].

Oxidative stress also plays a significant role in DED pathogenesis [88]. The ocular surface is vulnerable to oxidative damage from UV exposure, pollutants, and chronic topical treatments such as anti-glaucoma drops, all of which promote inflammation and oxidative stress [89,90]. Meta-analyses confirm increased oxidative markers, including lipid peroxides, malondialdehyde, ROS, and myeloperoxidase in tears and conjunctiva of DED patients compared to controls [88]. Age-related oxidative damage impairs lacrimal and meibomian gland function, destabilising the tear film [89]. Increased malondialdehyde and 4-hydroxy-2-nonenal levels highlight their potential as diagnostic biomarkers, though further validation is required [91].

Although antioxidant profiles in DED remain understudied, vitamin B12, L-carnitine, and selenoprotein P have demonstrated symptomatic benefits [89]. The Keap1-Nrf2/HO-1 antioxidant pathway is a promising therapeutic target, as its activation reduces oxidative damage and symptoms in a mouse model [92]. Additionally, the AMPK/ULK1 pathway, which regulates autophagy under oxidative stress, may alleviate DED when inhibited [93]. Proteomic analyses in Sjögren’s syndrome reveal decreased PRDX6 and CXCL1, key antioxidant and mucosal defence proteins correlated with disease severity [94]. These findings position oxidative stress as a central driver and promising therapeutic target in DED treatment.

#### 3.4.3. Mitochondrial Dysfunction in Ocular and Psychiatric Disease

Mitochondrial oxidative stress and mitochondrial DNA (mtDNA) damage are recognised as key contributors to the pathogenesis of DED [95]. Mitochondrial dysfunction disrupts energy production and increases ROS, causing apoptosis and the release of oxidised mtDNA into the cytosol. This activates the NLRP3 inflammasome and the cGAS–STING pathway, amplifying ocular surface inflammation. Inhibition of these pathways reduces the expression of inflammatory cytokines in DED conjunctival cells [96,98]. Sirtuin 1 (SIRT1), a mitochondrial regulator with antioxidant effects, is downregulated in DED, and SIRT1 activators show therapeutic potential [97]. Targeting mitochondrial dysfunction may interrupt the oxidative stress–inflammation cycle in DED [96].

Mitochondrial dysfunction is increasingly recognised as a key contributor to the pathophysiology of depression. Patients with depression exhibit reduced ATP production, impaired electron transport chain (ETC) activity, and altered mitochondrial gene expression, particularly in the hippocampus, contributing to fatigue and somatic symptoms [99]. Antidepressants such as fluoxetine and venlafaxine have been shown to restore mitochondrial function by enhancing ATP synthesis and antioxidant gene expression [99]. Oxidative damage further promotes the release of mtDNA, which triggers neuroinflammatory responses [100], and mtDNA variants associated with depression and anxiety may underlie elevated CRP levels and psychiatric symptomatology [101]. Additionally, stress-activated microglia exacerbate ROS production and mitochondrial impairment, thereby reinforcing neuroinflammation in depression [103]. Notably, ETC activity has been proposed as a predictor of antidepressant response, and mitochondrial dysfunction often precedes clinical onset of depression [102].

### 3.5. Serotonergic Signaling in Mood and Tear Regulation

Serotonin (5-hydroxytryptamine, 5-HT) is a key neuromodulator involved in mood, cognition, and neuroendocrine regulation. Synthesised mainly in the brainstem’s raphe nuclei and enteroendocrine cells, it modulates emotional processing, decision-making, and memory [104]. Dysregulated serotonergic signalling has been linked to depression, anxiety, PTSD, and OCD [105]. Its effects are mediated via seven receptor families comprising 14 subtypes, which regulate synaptic plasticity and neuronal excitability [106,107].

SSRIs are widely used for psychiatric disorders, based on the monoamine hypothesis of depression [105]. However, recent meta-analyses found no consistent link between reduced serotonin levels or serotonin-related genes and depression [108,109]. Moreover, acute serotonin depletion did not impair emotional regulation in healthy individuals, challenging its central role in mood control [110]. Emerging data suggest a context-dependent role involving circadian, genetic, and epigenetic mechanisms. Specifically, the circadian receptor REV-ERBα represses *Tph2*, the gene encoding tryptophan hydroxylase-2, thereby modulating depressive-like behaviour via the dorsal raphe nucleus in a preclinical study on mice [111].

While serotonin has been extensively studied in systemic disease, particularly gastrointestinal and central nervous system disorders [112], its role in ocular physiology, particularly in tear secretion and DED, remains underexplored. Tears form a three-layered protective film produced by different ocular structures: the lacrimal gland secretes the aqueous layer, Meibomian glands produce lipids, and conjunctival goblet cells release mucin. Tear production, especially the aqueous layer, is largely regulated by autonomic and sensory innervation [30,113,114]. Serotonin modulates aqueous secretion by enhancing noradrenaline release through presynaptic receptors [114]. Most serotonin receptors are G protein-coupled, except for 5-HT3, which is ionotropic [107]. Activation of 5-HT3a receptors in lacrimal gland acinar cells increases intracellular calcium, stimulating tear secretion [113].

Serotonin is present in the tear film, where its levels correlate with corneal sensitivity and peripheral sensitisation in DED [29,33]. Various serotonin receptors are expressed in ocular tissues, specifically 5-HT2A/2B in the retina, ciliary body, and conjunctiva, and 5-HT3, 5-HT4, and 5-HT7 in the conjunctiva, reflecting their diverse physiological roles [115,116]. In a Parkinson’s disease mouse model, eyelid serotonin levels fluctuate with disease progression, potentially altering tear composition and pain sensitivity, thus contributing to DED [117]. Elevated tear serotonin is associated with more severe symptoms [16]. Notably, individuals experiencing dry eye symptoms despite normal tear function exhibit heightened sensitivity to wind and light, indicating potential sensory dysregulation [29]. In a DED mouse model, activation of the 5-HT1A receptor exacerbates oxidative stress, inflammation, and corneal damage, whereas its inhibition confers protection [118]. These findings highlight serotonin’s emerging but underexplored role in DED pathophysiology.

## 4. Neurosensory Dysfunction and Central Pain Mechanisms

**Figure 1:**
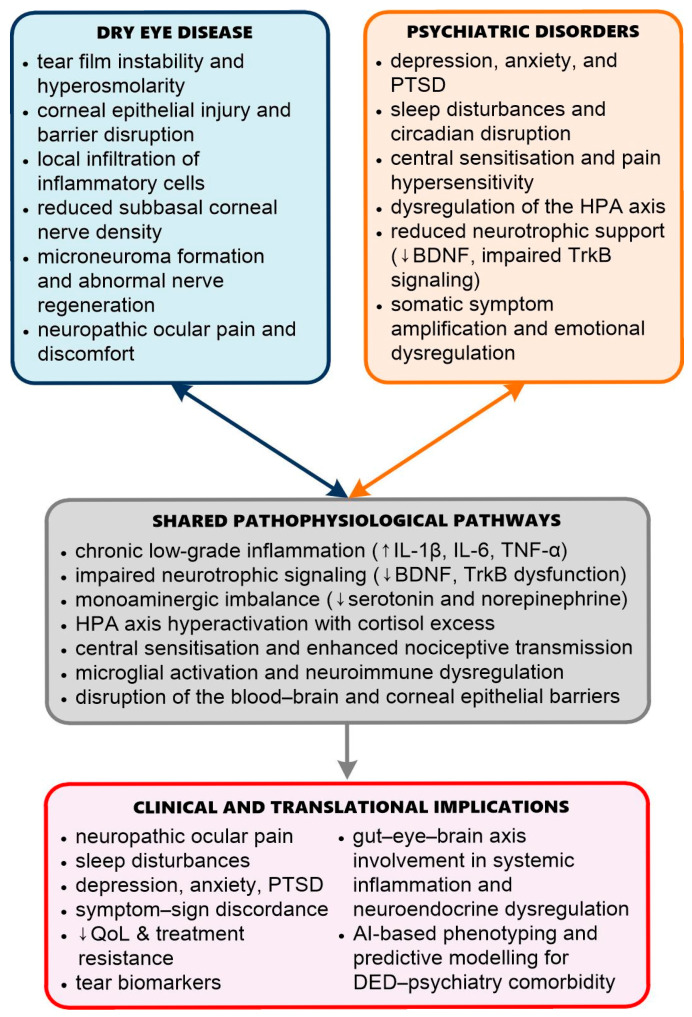
Shared neuroimmune and neuropathic pathways linking dry eye disease and psychiatric disorders. DED: Dry Eye Disease; PTSD: Posttraumatic Stress Disorder; HPA: Hypothalamic–pituitary–adrenal axis; BDNF: Brain-derived Neurotrophic Factor; Trk: tyrosine kinase; IL: interleukin; QoL: Quality of Life.

Emerging evidence suggests that the link between DED and psychiatric disorders extends beyond epidemiological associations and shared symptomatology, encompassing deeply interconnected neuroimmune and neuropathic mechanisms [4,5,6,7,8,9,10,11,12]. Figure 1 illustrates the conceptual framework of these shared pathways, highlighting how peripheral ocular surface inflammation and neurosensory alterations converge with central mechanisms of stress regulation, emotional processing, and pain modulation. While systemic inflammation, neurotransmitter dysregulation, and hormonal imbalances significantly contribute to both conditions, recent studies highlight the role of structural and functional changes in the peripheral and central nervous systems that further reinforce this bidirectional relationship [17,26,27,28,38,51,60]. Specifically, alterations in corneal innervation, dysregulated neuropeptide signalling, and sensitisation of the trigeminal–limbic pain axis create a complex neurobiological interface through which ocular and emotional symptoms interact [26,28,78]. These changes are paralleled by central mechanisms including microglial activation, HPA axis dysregulation, and impaired neurotrophic signalling, all of which are implicated in the pathophysiology of depression, anxiety, and chronic pain [51,52,53,70,74].

### 4.1. Corneal Nerve Alterations and Neuropathic Ocular Pain

Neurosensory dysfunction is a key contributor to the pathogenesis of DED. Reduced tear secretion and tear film instability lead to inflammation and damage of peripheral corneal nerves, which may result in neuropathic pain [126]. The cornea, particularly its subbasal nerve plexus, is densely innervated, and in vivo confocal microscopy enables visualisation of nerve alterations [30,31].

DED patients frequently exhibit reduced corneal nerve fibre density, increased tortuosity, and morphological changes consistent with nerve damage and regeneration [30,31,32]. These alterations correlate with symptoms such as hypoesthesia, dysesthesia, and heightened sensitivity, particularly in Sjögren’s syndrome [30]. Both main and branching nerve fibres are decreased in DED, with more pronounced reductions observed in aqueous-deficient subtypes compared to evaporative forms [32]. Aqueous-deficient DED is also associated with reduced epithelial cell density, corneal thinning, and marked stromal inflammation [127].

Increased nerve tortuosity, an indicator of aberrant nerve regeneration, is positively correlated with DED severity and negatively with tear film stability [31,128]. A meta-analysis confirmed reductions in corneal nerve length and density in DED patients but highlighted the need for further validation of IVCM as a diagnostic tool [128,129]. Microneuromas, localised swellings of subbasal nerve fibres, are frequently observed in patients with neuropathic corneal pain (NCP), especially in cases of DED linked to Meibomian gland dysfunction [126].

Depression and neuropathy are bidirectionally linked, with anxiety and depressive symptoms more prevalent in individuals with neuropathic pain, particularly those exhibiting severe symptoms and high levels of pain sensation [129]. Depressive symptoms are significantly more common in patients with diabetic neuropathy [130]. Shared pathophysiological mechanisms between depression and neuropathic pain include chronic psychosocial stress, systemic and neuroinflammation, autoimmune responses, and maladaptive neuroplasticity [131].

Neurotransmitter systems implicated in both conditions include monoamines and BDNF, with reduced BDNF levels and GABAergic dysfunction contributing to heightened pain sensitivity and depressive symptoms [132]. In the murine model, sciatic nerve injury induced depressive-like behaviours within eight weeks, accompanied by microglial activation and inflammation in the prefrontal cortex, hippocampus, and amygdala, regions central to mood regulation [133]. Accordingly, patients with DED and comorbid NCP are likely to exhibit more severe depressive symptoms [134].

### 4.2. Neuropeptides in Pain and Emotion

CGRP is a neuropeptide abundantly expressed in the peripheral and central nervous systems, particularly in the dorsal horn and trigeminal ganglion [18,135,136]. It plays a key role in pain transmission and central sensitisation, with elevated levels observed in various pain conditions [137]. CGRP is also found in brain regions involved in emotional processing, including the hippocampus, amygdala, hypothalamus, and thalamus [136]. Following nerve injury or inflammation, CGRP synthesis increases, partly via nerve growth factor (NGF) released by activated macrophages. Upon peripheral nerve stimulation, CGRP induces neurogenic inflammation through vasodilation [137].

Furthermore, a preclinical study shows that CGRP is also involved in mood regulation. In a murine stress model, reduced hippocampal CGRP mRNA levels were associated with depressive-like behaviour, whereas intracerebral CGRP administration before stress prevented these symptoms by enhancing NGF signalling [138]. Clinical evidence further indicates that anti-CGRP monoclonal antibodies may alleviate depressive symptoms in migraine patients, independent of headache relief; however, additional studies are required to confirm these findings [139].

In the ocular system, CGRP is released from corneal sensory nerves and stimulates lacrimal gland secretion, suggesting a protective role in tear production [140]. Dysregulated CGRP expression is noted in autoimmune DED, such as Sjögren’s syndrome and ocular cicatricial pemphigoid [12]. A recent study showed reduced tear levels of CGRP and substance P in DED patients, especially in severe cases, reinforcing the role of neuropeptides in DED pathophysiology. These neuropeptides, secreted from nerve endings and ocular surface tissues, contribute to inflammation, reflex tearing, and ocular discomfort, emphasising the importance of the neuroimmune interface in DED [34].

Transient Receptor Potential Vanilloid 1 (TRPV1) is a polymodal receptor expressed in small and medium-sized nociceptive neurons, including C- and Aδ-fibers of the dorsal root and trigeminal ganglia. In the cornea, TRPV1 is present on cold-sensitive nerve fibres, contributing to cold-induced pain and regulation of the tear reflex [35]. In DED, TRPV1 expression is upregulated in the trigeminal ganglion, enhancing cold allodynia and pain through the release of substance P [141]. Preclinical studies in adult male mice demonstrate that the TRPV1 antagonist capsazepine reduces corneal sensitivity to noxious stimuli, alleviates pain, and diminishes DED-related anxiety, highlighting its therapeutic potential [142]. While substance P promotes corneal epithelial healing, its overexpression induces hyperalgesia, inflammation, and neovascularisation. Altered substance P levels in DED and its interaction with the neurokinin-1 receptor (NK1R) underscore NK1R antagonists as a promising target for future therapeutic strategies [33].

TRPVI is increasingly recognised for its role in higher brain functions, including learning, memory, and emotional regulation [143]. TRPV1-deficient mice exhibit reduced fear and anxiety; however, the effects of TRPV1 modulation in humans remain poorly understood [144]. In mice fed a high-fat diet, depressive-like behaviour has been linked to TRPV1 dysfunction in brain regions such as the prefrontal cortex, hippocampus, hypothalamus, and amygdala [145]. Preclinical studies in mice and rats further demonstrate that TRPV1 antagonism in the hippocampus and amygdala produces anxiolytic and antidepressant effects, likely through enhanced serotonergic activity [143].

TRPV1 also contributes to neuropathic pain through microglial activation and glutamatergic signalling at the synaptic level. However, conflicting evidence suggests that TRPV1 may not modulate synaptic plasticity in the anterior cingulate cortex, as demonstrated in adult male mice [146]. Substance P, a key mediator of pain and emotion, is released during nociceptive signalling and amplifies pain via NMDA receptor sensitisation. Elevated substance P levels have been observed in individuals with depression and anxiety, and are associated with structural brain changes and hyperalgesia [147]. Acting through NK1 receptors, substance P contributes to mood and anxiety disorders, making NK1 receptor antagonists a promising therapeutic strategy for depression, anxiety, and PTSD, as suggested by findings from a limited number of clinical trials [148].

### 4.3. Trigeminal–Limbic Pathways and Central Sensitisation

Central sensitisation refers to heightened pain perception caused by repeated activation of peripheral nociceptors, particularly C-fibres, leading to excessive NMDA receptor activation in the dorsal horn of the spinal cord and reduced pain thresholds [5,64,149,150]. This mechanism is closely linked to depressive disorders through limbic system involvement. Pain activates the kappa opioid system, which reduces dopamine release in the nucleus accumbens, a pathway implicated in the emotional burden of chronic pain and the development of depressive symptoms [5,64].

In DED, nociceptive pain arises from ocular surface stimuli such as tear film instability and inflammation, while neuropathic pain results from dysfunction in the sensory nervous system. Corneal pain is mediated by Aδ and C fibres of the ophthalmic branch of the trigeminal nerve, with cell bodies in the trigeminal ganglion projecting to the spinal trigeminal nucleus [149,150]. From there, pain signals ascend to the thalamus and somatosensory cortex, where they are integrated within the limbic system, including the amygdala, insula, anterior cingulate cortex, and prefrontal cortex, regions responsible for the emotional aspect of pain [149,150].

Central sensitisation in DED often involves hyperexcitability of neurons in the subnucleus caudalis of the spinal trigeminal complex [150,151]. Glutamatergic transmission via AMPA, NMDA, and mGluR5 receptors enhances pain signalling, while reduced GABAergic inhibition contributes to persistent hypersensitivity. Supporting this mechanism, a preclinical study in rats demonstrated that activation of GABA A receptor agonists may reduce pain in this region, highlighting the role of excitation/inhibition imbalance in chronic ocular pain [150].

Resting-state fMRI studies in patients with DED reveal abnormal regional neural activity (ReHo) within limbic–cortical circuits, supporting a link between central neural alterations, chronic pain, and mood disorders [151]. Central sensitisation is further exacerbated by microglial activation and neuroinflammation [152]. Persistent ocular nerve injury induces changes in the expression of ion channels and receptors, including NMDA receptors, within the trigeminal system, promoting the transition to neuropathic pain. Consistently, a preclinical study on rats indicates that NMDA receptor antagonists may show therapeutic potential in alleviating centrally mediated ocular pain [153].

These mechanisms explain why DED patients may report severe discomfort despite normal clinical findings and why subjective symptoms often correlate more strongly with psychiatric comorbidities than with objective ocular signs [17]. Elevated serotonin levels in tears may serve as a biomarker of peripheral pain sensitivity in DED, but not in cases dominated by central sensitisation [29].

### 4.4. Microglial Activation and Neuroinflammation

Psychosocial stress in depression activates the NLRP3 inflammasome via endogenous damage-associated molecular patterns (DAMPs), triggering the release of pro-inflammatory mediators that stimulate microglial activation [51,60]. Activated microglia secrete prostaglandins, leukotrienes, and chemokines, such as CXCL4, CXCL7, and CXCL8, that promote monocyte recruitment and sustain a neuroinflammatory feedback loop contributing to neuronal damage, particularly in the limbic system [60]. Elevated levels of Iba1, a marker of microglial activation, are commonly observed in depression. Chronic glucocorticoid exposure further amplifies NLRP3 inflammasome activity and microglial proliferation, linking prolonged stress to immune and affective dysregulation [70].

Monoaminergic dysfunction in depression also contributes to excessive glial activation [60,71]. Preclinical rat studies have demonstrated that norepinephrine exerts anti-inflammatory effects via β-adrenergic receptors on glial cells. Serotonin effects are concentration- and receptor-dependent: 5-HT1A and 5-HT7 receptor activation is anti-inflammatory, whereas 5-HT2A/2B/2C receptors promote microglial activation and NF-κB signalling [71]. Additionally, mouse models indicate that activation of astrocytic dopamine D2 receptors suppresses inflammation through alpha B-crystallin (CRYAB)-mediated pathways, whereas receptor antagonism enhances glial activation and inflammation [71]. These findings underscore the critical role of glial–immune interactions in the pathophysiology of mood disorders [51,60,70,71].

Functional parainflammation is a protective, regulated immune response mediated by factors such as Nrf2 and NF-κB, which promote the production of antioxidants and growth factors, thereby maintaining homeostasis [154]. However, when such inflammation becomes chronic, it contributes to diseases such as glaucoma and age-related macular degeneration (AMD) [154]. In DED, persistent inflammation reflects dysregulated immune mechanisms that involve both innate and adaptive responses [155]. Neuronal release of proinflammatory mediators activates microglia and astrocytes, which then release cytokines, including IL-1β, IL-6, TNF-α, chemokines, BDNF, and excitatory molecules such as glutamate and ATP, leading to increased pain sensitisation [150]. Microglia are activated first, followed by prolonged astrocyte activation, which sustains neuropathic pain in DED [156]. In a mouse model with lacrimal gland excision, enhanced corneal sensitivity, trigeminal synaptic activity, elevated inflammatory markers, and increased expression of glial markers (Iba1, GFAP, CD68, Itgam) further confirmed the role of neuroinflammation in pain generation [157]. Other mouse models similarly demonstrate heightened microglial activity in the trigeminal nucleus, which subsides once corneal inflammation resolves, indicating reversibility of both peripheral and central sensitisation [152]. Glial activation also contributes to retinal diseases such as AMD through the activation of microglia and the complement system [154]. Collectively, these findings indicate that neurogenic inflammation, mediated by both peripheral and central sensitisation, plays a key role in the pathophysiology of DED [150,154,156,157].

## 5. Psychotropic Medications and Ocular Surface Health

Psychotropic medications, including TCAs, SSRIs and SNRIs, benzodiazepines, pregabalin, gabapentin, antipsychotics, and mood stabilisers, can compromise ocular surface health through anticholinergic activity, tear film destabilisation, and altered corneal innervation [158,159,160,161]. The eye’s rich vascular supply facilitates systemic drug penetration, making ocular side effects relatively common [157]. Many of these agents are associated with measurable declines in tear production or stability, pro-inflammatory changes, and structural alterations in the cornea, underscoring the need for vigilance in patients with pre-existing DED [158,159,160,161].

Table 2 summarises the ocular surface impact of frequently prescribed psychotropic medications, including mechanisms, clinical symptoms, and evidence-based management strategies [46,76,157,158,159,160,161,162,163,164,165,166,167,168,169,170,171,172,173,174,175,176,177,178,179,180].

### 5.1. Antidepressants and Anxiolytics

TCAs exert potent anticholinergic effects, leading to reduced lacrimal and goblet cell secretion, and are frequently associated with mydriasis, blurred vision, and an increased risk of acute angle-closure [158,159]. Although SSRIs and SNRIs possess weaker anticholinergic properties, they have been associated with elevated intraocular pressure and, in rare cases, acute glaucoma [158,159]. Benzodiazepines, pregabalin, and gabapentin may cause blurred vision, diplopia, or acute glaucoma episodes [159].

Multiple studies have demonstrated that antidepressants and anxiolytics significantly increase the risk of developing DED, independent of the presence of depression [158,160,161]. Large population-based analyses rank the use of these agents as a DED risk factor on par with glaucoma, blepharitis, and the use of antihypertensive medications [161]. TCAs cause the greatest reduction in Schirmer I test scores, whereas SSRIs reduce both Schirmer and Tear Break-up time Test (TBUT) values. In contrast, SNRIs primarily decrease TBUT, indicating impaired tear film quality without significant loss of aqueous volume [158,162]. Although anticholinergic mechanisms are predominant, a rat study by Dankis et al. demonstrated that even agents with minimal anticholinergic activity, such as escitalopram, can suppress reflex tear secretion, suggesting that disrupted neural regulation also contributes to the pathophysiology of DED [163].

SSRIs may further impair ocular surface integrity by modulating tear serotonin concentrations and activating NF-κB–mediated inflammation [158,164]. In one study, 75% of individuals receiving SSRIs exhibited a TBUT of less than 10 s accompanied by corneal epithelial damage, despite normal Schirmer test values, indicating a selective impairment of tear film stability rather than aqueous tear production [159]. SNRIs such as duloxetine and venlafaxine may reduce ocular pain perception through central pain-modulation pathways [158,159]; however, elevated IL-17 and TNF-α in tears of depressed DED patients suggest a pro-inflammatory milieu influenced by drug selection [72]. While dry eye is typically labelled a rare adverse effect, clinicians should consider drug class, anti-inflammatory properties, and neural impact when managing patients with coexisting psychiatric disease and DED [160].

### 5.2. Antipsychotics

Antipsychotic medications, particularly atypical agents, are increasingly recognised as contributors to ocular surface alterations, including reduced tear secretion, tear film instability, epithelial damage, corneal thinning, and pigment deposition [165]. Severe DED can develop within the first 1–5 years of treatment, with prevalence up to 20% after one year [166]. Antipsychotic-induced neuromuscular effects, such as blepharospasm and tics, reported with second-generation agents, can exacerbate evaporative DED [167].

Many antipsychotics’ anticholinergic effects contribute to tear film dysfunction [20,168]. Clozapine, a strong muscarinic M3 receptor antagonist critical for tear secretion [169,170], is especially implicated, alone or with quetiapine [168,170]. It is associated with decreased corneal thickness and markedly reduced Schirmer and TBUT scores compared to controls [164,171]. Clozapine’s antagonism of 5-HT2C receptors also lowers blink rate by suppressing nigrostriatal dopamine, further impairing tear film renewal and worsening dry eye [171].

Importantly, DED often goes undiagnosed in patients with schizophrenia due to their typically minimal subjective symptoms [165]. Consequently, clinicians should maintain a high level of vigilance, particularly in cases of polypharmacy and long-term antipsychotic use, and consider routine ophthalmologic screening for this vulnerable group.

### 5.3. Mood Stabilisers

Mood stabilisers such as lithium and valproate have been linked to tear film instability and the development of evaporative DED [46,172]. Lithium, which is excreted in tears, increases tear osmolarity and reduces tear production, causing ocular irritation [173,174]. Rarely, lamotrigine may trigger Sjögren’s-like symptoms through autoimmune pathways involving IL-4 and IL-5 [175]. Similarly, lithium may promote autoimmunity in salivary glands, while valproate induces oxidative stress, damaging lacrimal glands [173,176]. Although lithium does not affect the blink reflex, its ocular side effects are often underrecognized, potentially leading to treatment nonadherence [173,174]. Current evidence on antiepileptic and mood stabilisers in DED is limited to small studies and case reports, highlighting the need for further research [177]. Notably, longer duration of psychotropic use is consistently associated with higher DED prevalence across antidepressants, anxiolytics, and antipsychotics [166,178].

### 5.4. Polypharmacy and Cumulative Risk

Polypharmacy, typically defined as the use of five or more medications, is a significant iatrogenic risk factor for DED, especially in older adults, increasing the likelihood of ocular or oral dryness by 88% [179]. The additive anticholinergic burden and neurotransmitter interactions can impair parasympathetic regulation of lacrimal and salivary secretion, a critical consideration in suspected Sjögren’s syndrome, where medications may mimic or exacerbate glandular dysfunction [180]. Along with systemic diseases and age-related structural changes, polypharmacy is a leading cause of DED in the elderly [181]. Moreover, antipsychotic polytherapy confers a higher risk of DED compared to monotherapy [168], underscoring the importance of thorough medication review to optimise ocular surface health.

Although it remains challenging to clearly differentiate primary from secondary, psychopharmacologically induced DED, the existing literature allows for a pragmatic clinical distinction [76,158,159,160,161,162,163,164,165,166,167,168,169,170,171,172,173,174,175,176,177,178,179,180,181]. Secondary DED is typically associated with the use of medications that impair the blink reflex, reduce lacrimal secretion, or disrupt ocular surface homeostasis. This is particularly evident in patients receiving polypharmacy, especially drugs with anticholinergic properties or long-term psychotropic therapy. In such cases, additional ocular side effects, including blurred vision, pupillary changes, or ocular surface toxicity, are often present alongside DED manifestations [158,159,160,161,162,163,164,165,166,167,168,169,170,171,172,173,174,175,176,177,178,179,180,181].

## 6. Clinical and Translational Perspectives

In some patients, severe dry eye symptoms may primarily reflect underlying psychological distress rather than ocular surface damage. If overlooked, this can result in ineffective, surface-focused treatment. Routine screening for depression and anxiety in DED is essential, as comorbid psychological disorders can impair treatment response and prolong symptoms [27]. Integrated care between ophthalmologists and mental health professionals supports personalised management and improves outcomes [4].

Severe symptoms may also indicate neuropathic pain, often associated with chronic illness, central sensitisation, and overlapping pain conditions [69,182]. These patients may not benefit from standard therapies and may require treatments targeting neural dysfunction. Corneal neuropathic pain often mimics severe DED, leading to potential misdiagnosis.

Emerging therapies targeting corneal nerve dysfunction include topical nerve growth factor (NGF), already approved for neurotrophic keratitis, and may aid in regenerating damaged corneal nerves in CNP. Other promising drugs include lacosamide, low-dose naltrexone, enkephalin modulators, and libvatrep [183,184]. Accurate diagnosis of psychogenic or neuropathic DED requires careful history-taking, awareness of neuropathic symptoms such as burning, allodynia, refractory pain, and advanced imaging, such as in vivo confocal microscopy [183,185].

### 6.1. Tear-Based Biomarkers for Diagnosis and Monitoring

Tear fluid contains cytokines, chemokines, and growth factors that indicate ocular surface inflammation and disease severity, providing key insights for diagnosing and monitoring DED [186]. Tears are readily accessible and can be collected noninvasively, making them ideal for biomarker analysis. Inflammatory markers such as IL-1β and IL-6 indicate epithelial damage, while EGF reflects the function of the lacrimal gland [29,112,186,187]. Tear biomarkers are more sensitive and correlate better with disease severity than traditional tests like Schirmer’s and corneal staining. Their noninvasive nature and easy accessibility make tears an attractive medium for clinical assessment. Despite this, their routine use is limited by high costs, sampling variability, diurnal fluctuations, low specificity, and inherent biological heterogeneity [29,112,187]. Notably, tear composition is influenced by sex, as sex steroids differentially modulate tear secretion [114]. In addition, standardised reference ranges for key molecules, including serotonin, remain unavailable across different body fluids [112]. To overcome these limitations, the establishment of standardised sampling protocols and the development of composite biomarker indices are critical, promising to enhance both the accuracy and translational potential of tear-based diagnostics [187].

### 6.2. Emerging Molecular and Neuromodulatory Therapies

Emerging treatments targeting inflammatory and neuroregenerative pathways show promise for DED. The JAK-STAT pathway, activated by cytokines such as IL-2, IL-6, IL-17, and TNF-α, along with the SYK, which facilitates antigen presentation and immune cell recruitment, represents a key mediator of ocular surface inflammation [188,189]. In preclinical models, topical JAK inhibitors, such as tofacitinib, have demonstrated notable anti-inflammatory effects, including reduced expression of conjunctival HLA-DR. However, authors emphasise that larger clinical trials are needed to confirm these findings and establish therapeutic efficacy [188,189].

Recombinant human nerve growth factor (rhNGF, Cenegermin), approved for neurotrophic keratitis, promotes epithelial healing, nerve regeneration, tear secretion, and tear film stability, making it a promising option for severe DED [190]. ST-100, a collagen-mimetic peptide, supports corneal nerve regeneration and ECM repair, particularly in patients with corneal hypoesthesia [191].

Targeting TRPV1 also offers therapeutic potential by reducing aberrant pain signaling and associated discomfort, thereby addressing the emotional component of chronic ocular pain [142,192]. TRPV1 antagonists, such as capsazepine, show promise for neuropathic DED, while broader TRP channel modulators enhance tear secretion, increase tear meniscus height, reduce conjunctival hyperemia, and improve both quality of life and daily functioning [193].

### 6.3. Multidisciplinary and Lifestyle Interventions

A multidisciplinary approach is increasingly recognised as essential for effective DED management. In the mouse model, combined treatment with the anti-inflammatory lifitegrast and antioxidant tocopherol outperformed monotherapy by reducing inflammation, oxidative stress, and corneal damage while improving tear volume and stability [194]. A 10-week exercise program integrating cognitive-behavioural therapy also improved dry eye symptoms and psychological well-being in healthy office workers, highlighting the importance of lifestyle and stress management [195].

New therapies targeting neurosensory pathways show promise in DED. Varenicline nasal spray, acting on nicotinic receptors of trigeminal nerves, significantly increased tear production over 12 weeks in clinical trials without serious adverse effects [196]. In a rabbit study, topical gabapentin demonstrated analgesic, anti-inflammatory, and tear-enhancing effects, likely through increased expression of acetylcholine, norepinephrine, and aquaporin 5 in lacrimal glands [197]. A cross-sectional retrospective study suggests that botulinum toxin A (BoNT-A) alleviates migraine, photophobia, and dry eye symptoms by modulating CGRP-mediated neural pathways [198].

## 7. Integrated Management Strategies

DED is a multifactorial disease involving tear instability, inflammation, neurosensory dysfunction, and psychological factors. Effective treatment requires a comprehensive, personalised approach that addresses both ocular and systemic causes, particularly when symptoms exceed clinical findings [1,2,3,4].

### 7.1. Personalised Approaches and Phenotyping

DED exhibits considerable heterogeneity, with some patients experiencing severe symptoms despite minimal signs [23,44]. Psychiatric comorbidities and the use of psychotropic medications can further destabilise the tear film and exacerbate ocular surface inflammation, contributing to persistent symptoms and suboptimal therapeutic outcomes [27,44,199,200].

A stepwise, personalised approach should begin with conventional therapies including preservative-free artificial tears, topical anti-inflammatories such as cyclosporine A or lifitegrast, and environmental adjustments to reduce evaporative stress [201]. As discussed, emerging neuromodulatory treatments, including topical nerve growth factor (NGF), TRPV1 antagonists, and nicotinic agonists, such as varenicline nasal spray, show promise in restoring corneal innervation and enhancing tear secretion [190,192,196].

In cases of neuropathic ocular pain, pharmacological agents such as gabapentinoids, low-dose naltrexone, or botulinum toxin A may reduce pain sensitivity and modulate aberrant trigeminal signalling [183,184,197,198]. When symptoms and signs differ markedly, mental health screening tools such as Patient Health Questionnaire-9 (PHQ-9) and Generalised Anxiety Disorder-7 (GAD-7) can support identification of psychological distress [202,203]. Referral to mental health specialists is advised if psychiatric factors impair treatment response. This integrated approach targets both ocular and central emotional factors, enhancing therapeutic effectiveness and long-term outcomes.

### 7.2. Ocular Impact of Psychotropic Medications

The association between DED and psychiatric disorders is well established. Shared mechanisms likely involve immune dysregulation, chronic inflammation, and genetic pleiotropy, as supported by genome-wide association studies [204].

Combining standard DED treatments with psychiatric therapy, pharmacological or psychotherapeutic, can improve symptom control and overall well-being [200]. However, many psychotropic drugs, particularly those with anticholinergic or sympathomimetic effects, may worsen DED by reducing tear secretion and damaging the ocular surface [44,158,159,160,161]. This risk increases with polypharmacy, underscoring the need for careful medication review and close collaboration between ophthalmologists and mental health professionals to optimise care [5,9].

### 7.3. Patient Education, Adherence and Psychosocial Support

Effective DED management extends beyond pharmacotherapy, requiring patient education, adherence, and psychosocial support. Patients should be informed about non-modifiable risk factors, including age, female sex, East Asian ethnicity, as well as modifiable behaviours such as screen overuse, poor sleep, contact lens wear, and exposure to low humidity [205,206,207].

Education enhances adherence, sets realistic expectations, and supports lifestyle changes. Chronic ocular discomfort can severely impact quality of life, particularly in vulnerable groups like those with Sjögren’s syndrome [7,20,134]. Integrating psychological support and routine mental health screening enables more holistic, patient-centred DED care [134].

### 7.4. Integrated Ophthalmologic–Psychiatric Care in Dry Eye Disease

Symptom–sign discrepancy in DED often reflects underlying psychological factors such as anxiety, emotional dysregulation, or somatisation [23,44,200], which, if unaddressed, can lead to persistent discomfort, poor adherence, and reduced treatment efficacy.

Integrated care between ophthalmologists and psychiatrists is essential, involving routine mental health screening, awareness of psychotropic ocular effects, and timely referral. Unaddressed psychological distress can amplify symptoms and promote central sensitisation [23,44]. Interdisciplinary coordination supports early detection of comorbidities, personalised treatment, and better quality of life. By targeting both peripheral and central drivers, it offers a more effective, holistic approach to managing DED in patients with psychiatric comorbidities [207].

## 8. Clinical Implementation and Care Pathways

Recognising the bidirectional link between DED and psychiatric disorders is essential for effective, patient-centred care. Psychiatric comorbidities such as depression, anxiety, PTSD, and sleep disturbances are common in DED and amplify symptom perception, impair adherence, and reduce treatment response [4,5,6,7,8,9,10,11,12,13,16,17,23,27,44]. Integrating ophthalmologic and psychiatric assessment enables early identification of these comorbidities and guides more personalised therapy. Routine use of brief screening instruments (e.g., PHQ-9, GAD-7) is recommended, particularly in patients who exhibit symptom–sign discrepancy or poor response to conventional treatment [13,23,44,207]. Interdisciplinary collaboration facilitates timely referral, optimises psychotropic medication choice, and enhances overall outcomes.

Emerging translational tools, including tear-based biomarkers (serotonin, BDNF, IL-6, TNF-α), offer potential for early diagnosis, patient stratification, and monitoring of treatment response. Combined with multimodal imaging and psychological profiling, they may support precision medicine approaches that bridge ophthalmology and psychiatry [29,51,55,60,70,81,84,88,95,99]. Implementing integrated care pathways, linking ophthalmologists, psychiatrists, and primary care providers, can improve quality of life, reduce chronicity, and address the full biopsychosocial spectrum of DED.

## 9. Future Perspectives

Despite increasing evidence supporting the interplay between DED and psychiatric disorders, significant knowledge gaps remain [5,6,7,8,9,14,16,17,20,38,44]. To translate current insights into clinical practice, several key areas warrant further investigation. Table 3 outlines strategic research directions aimed at advancing our understanding and management of DED, as well as its intersection with psychiatric disorders [4,6,7,13,14,15,16,23,27,29,30,31,32,33,41,44,53,55,56,58,72,112,121,122,151,153,200,207,208,209,210,211,212,213,214,215,216,217].

From a translational perspective, there is great promise in the development of tear-based biomarkers such as BDNF, serotonin, IL-6, TNF-α and cortisol. These biomarkers may help stratify patients, predict disease severity, monitor treatment response, and guide personalised therapeutic approaches. The integration of tear proteomics and metabolomics into clinical practice may eventually enable point-of-care diagnostics that bridge the ophthalmologic and psychiatric domains [16,29,33,56,58,72,121,122,186,187,208].

Future research should prioritise large-scale, longitudinal cohort studies to clarify the temporal dynamics and causal relationships between DED and psychiatric disorders. Most current studies are cross-sectional, limiting the ability to determine whether psychological symptoms precede ocular changes or arise as a consequence of chronic ocular discomfort [6,7,8,9,10,11,16,17,41,42,209]. Elucidating this directionality is essential for timely prevention, risk stratification, and early intervention strategies. Standardised diagnostic tools, both ophthalmological (such as tear osmolarity, IVCM, and tear proteomics) and psychiatric (including clinician-administered diagnostic interviews), should be employed consistently across studies to enable meaningful comparisons and facilitate subgroup analysis [1,3,4,39,44,55,210]. Combining these modalities may significantly enhance early detection of high-risk phenotypes and inform tailored therapeutic approaches.

Moreover, future investigations should explore neuroimaging correlates of central sensitisation in DED, particularly using fMRI to assess alterations in limbic and pain-processing circuits. Parallel studies of corneal nerve structure using IVCM and brain activity may illuminate novel neuro-ocular signatures of comorbidity. Combining in vivo confocal microscopy, tear proteomics, optical coherence tomography, and standardised psychiatric assessment tools could improve early detection and stratification of high-risk patients [13,30,31,32,44,126,127,128,129,151,210].

Furthermore, establishing composite biomarker panels and threshold values for clinical decision-making may advance the field toward precision medicine. Tear fluid, as a non-invasive and information-rich biological sample, represents a unique translational bridge between ocular surface disease and central nervous system dysfunction [16,29,33,56,58,72,121,122,186,187,208].

Emerging evidence highlights the significance of the gut–eye–brain axis in regulating both ocular surface homeostasis and neuropsychiatric function. Gut microbiota dysbiosis can disrupt systemic and central immune responses through the production of microbial metabolites, including short-chain fatty acids, tryptophan catabolites, and lipopolysaccharides. These molecules influence HPA axis activity, alter blood–brain barrier permeability, and modulate cytokine release, thereby contributing to both dry eye symptoms and psychiatric morbidity. In particular, gut-derived signals affect central serotonin and GABA pathways, linking microbial composition to emotional regulation, pain perception, and ocular surface inflammation. Furthermore, recent studies suggest that microbiota-driven immune dysregulation may impair lacrimal gland function and promote tear film instability, reinforcing the gut’s role in ocular disease [53,112,211,212,213,214].

In parallel, artificial intelligence (AI) and digital phenotyping are emerging as powerful tools for early detection, stratification, and personalised management of DED-psychiatric comorbidity. Machine learning models trained on multimodal datasets, including tear biomarker profiles, neuroimaging, patient-reported outcomes, and wearable sensor data, can uncover novel phenotypic clusters, predict therapeutic response, and support precision diagnostics. Smartphone-based passive monitoring and natural language processing of subjective symptom reports further enable real-time detection of emotional distress in DED patients. Integrating AI technologies with molecular diagnostics and brain–ocular correlates may accelerate the development of predictive models and multidisciplinary treatment strategies [207,215,216,217].

At the therapeutic level, interventions targeting shared mechanisms, such as anti-inflammatory agents with central effects, neurotrophic modulators, and antioxidant strategies, hold promise for dual benefit. Clinical studies should evaluate whether therapies targeting one domain, such as antidepressants and psychotherapy, affect outcomes in the other (ocular pain, inflammation), and vice versa. Agents that modulate the BDNF–TrkB pathway or regulate serotonin signalling may simultaneously alleviate ocular and psychiatric symptoms. Clinical trials assessing such agents should include ocular endpoints as secondary outcomes [28,60,75,76,160,188,189,190,191,192,193,194,195].

Clinically, a multidisciplinary model of care is essential. Ophthalmologists should routinely screen for psychiatric comorbidities in refractory DED cases, particularly when symptoms outweigh clinical findings. Mental health professionals should consider DED a potential somatic manifestation of mood disorders or a side effect of therapy. Patient-centred communication and cross-speciality collaboration are key to improving outcomes [6,7,13,15,23,27,30,41,44,55,207,210].

Finally, public health strategies should emphasise awareness campaigns, clinical guidelines, and patient empowerment initiatives to promote early screening, reduce diagnostic delays, and encourage interdisciplinary care. Specific at-risk populations such as postmenopausal women, patients with Sjögren’s syndrome, patients with PTSD, and elderly individuals on multiple medications should be prioritised for screening and multidisciplinary intervention [1,4,23,26,36,44,167,205,207,210].

While future research directions highlight promising avenues for translational and clinical advancement, several limitations of the present review should be acknowledged. As a narrative synthesis, this review is inherently qualitative and cannot establish causal relationships; instead, it outlines associations and mechanistic hypotheses linking DED and psychiatric disorders. Many cited studies are preclinical, cross-sectional, or based on small cohorts, which limits the generalizability of the findings. Consequently, distinctions between correlation, causation, and theoretical assumptions are sometimes unclear.

Research at the interface of psychiatry and ocular health is also susceptible to selection bias and relies heavily on subjective symptom reporting, while the lack of standardised diagnostic criteria for both DED and psychiatric conditions further restricts comparability across studies. Evidence regarding tear biomarkers, particularly serotonin and BDNF, remains preliminary, largely stemming from preclinical or small-sample investigations. Inconsistent sampling protocols and diurnal variability further complicate interpretation. Moreover, human translational data are still limited relative to preclinical findings, and the differentiation between primary DED and secondary DED induced by psychopharmacological treatments has not been adequately addressed. Future research should therefore prioritise large, longitudinal, and well-controlled clinical and translational studies that employ standardised methodologies to clarify causal mechanisms and enhance the clinical relevance of neuroimmune and neuropsychiatric findings in DED.

## 10. Conclusions

DED should be recognised not merely as a local ocular disorder but as a systemic condition with profound neuroimmune, psychosomatic, and psychiatric dimensions. Adopting a systems-level, biopsychosocial model that integrates ophthalmology and psychiatry provides a promising framework for both clinical practice and scientific progress. Routine mental health screening, interdisciplinary collaboration, and patient-centred communication can enhance diagnostic precision, optimise therapeutic outcomes, and substantially improve quality of life. Translating molecular and neuroimmune insights into patient-specific interventions remains crucial. Shared mechanisms, including systemic inflammation, HPA axis dysregulation, oxidative stress, mitochondrial dysfunction, and reduced BDNF expression, represent both pathophysiological drivers and therapeutic targets. Ultimately, bridging ophthalmology and psychiatry through integrated research and clinical innovation will foster the development of targeted, multimodal strategies that address both peripheral and central disease pathways. Such an integrated approach has the potential to transform patient care and improve quality of life in this complex and frequently under-recognised patient population. Recognising dry eye disease as a systemic, neuroimmune-driven disorder with strong psychiatric interplay underscores the need for integrated, multidisciplinary approaches that bridge ophthalmology and psychiatry in both research and patient care.

## Figures and Tables

**Table 1:** Shared Molecular and Neuroimmune Mechanisms Linking Dry Eye Disease and Psychiatric Disorders.

**Mechanism**	**DED-Related Findings**	**Psychiatric Disorder Findings**	**Shared Pathways**
HPA Axis Dysregulation	Tear cortisol levels correlate with dry eye symptoms Higher levels of salivary cortisol in patients with Sjögren’s syndrome Tear cortisol is a potential biomarker for DED-related symptoms Stress prolongs tear film instability and inflammation	Chronic stress leads to prolonged activation of the HPA axis Chronic HPA axis activation → ↑ cortisol, ↑ CRH → neuronal dysfunction, reduced neurogenesis, decreased dendritic density, mood dysregulation, sleep disturbances Sleep disturbances—mediators in the relationship between HPA axis dysregulation and DED symptoms	Stress-induced neuroinflammation Glucocorticoid receptor sensitivity Altered immune control
Monoaminergic Dysregulation (5-HT, NE, DA)	Disrupted monoaminergic signaling linked to altered pain perception in DED Higher tear serotonin levels in DED patients with symptoms and reduced tear production	Monoaminergic pathways modulate central pain in chronic pain and depression Monoamines regulate neuroplasticity and pain modulation across brain regions Reduced dopamine, serotonin, and noradrenaline levels in synapses of depressed patients Altered reuptake in anxiety and PTSD Norepinephrine projections regulate affective components of pain perception	Dysregulation of monoaminergic signaling contributes to altered pain perception in both DED and depression Monoamines play key roles in neuroplasticity and affective pain regulation Shared pathways influence ocular discomfort in DED and central sensitisation in psychiatric comorbidities
Proinflammatory Cytokines	DED—inflammatory condition Increased IL-1β, IL-6, IL-17 and TNF-α in conjunctiva and tears High levels of cytokines correlate with symptom severity and the progression of DED	Chronic stress is a key trigger of low-grade systemic inflammation Elevated proinflammatory cytokines (IL-6, IL-1β, IL-10, TNF-α) indicate pronounced neuroinflammation Cytokines (IL-2, IL-2R, IL-4, IL-10, TGF-β) and CRP are associated with the development and severity of depression Proinflammatory cytokines impair neurotransmitter signaling and glucocorticoid regulation Neuroinflammation leads to reduced neurogenesis (via the IL-1β–induced kynurenine pathway), glutamate excitotoxicity (mediated by TNF-α), and increased blood–brain barrier permeability Inflammatory markers serve as indicators of depression severity and predictors of treatment response SSRIs and SNRIs reduce proinflammatory and increase anti-inflammatory cytokine levels Anti-inflammatory and immunomodulatory therapies show efficacy in alleviating depressive symptoms	Chronic stress → low-grade systemic inflammation ↑ IL-6, IL-1β, IL-10, TNF-α → neuroinflammation and ocular surface inflammation ↑ IL-2, IL-2R, IL-4, IL-10, TGF-β, CRP → linked to symptom severity in both conditions Cytokines disrupt neurotransmission and HPA axis regulation IL-1β activates the kynurenine pathway → ↓ neurogenesis TNF-α → glutamate excitotoxicity and ↑ blood–brain barrier permeability Inflammatory markers predict disease severity and treatment response SSRIs/SNRIs → ↓ proinflammatory and ↑ anti-inflammatory cytokines Anti-inflammatory and immunomodulatory therapies beneficial for both mood and ocular symptoms
Oxidative Stress and Mitochondrial Dysfunction	UV radiation, pollution, and anti-glaucoma drugs → ↑ oxidative stress in ocular tissues ↑ Oxidative stress markers (MDA, 4-HNE) in tears and conjunctiva Activation of Keap1–Nrf2/HO-1 pathway reduces oxidative damage and DED symptoms	↑ Oxidative stress and systemic inflammation damage neurons ↓ BDNF levels due to oxidative imbalance ↑ Oxidative markers: ↓ antioxidant levels in depression ↓ ATP production and electron transport chain dysfunction SNPs in mitochondrial DNA linked to anxiety and depression Depression may precede mitochondrial disease diagnosis MeCP2 dysregulation implicated in depression Reactive nitrogen species (RNS) contribute to neuronal injury	Oxidative stress as a central driver in both DED and depression ROS and RNS damage shared cellular targets (lipids, proteins, DNA) Mitochondrial dysfunction contributes to neurodegeneration and ocular surface damage Nrf2/HO-1 pathway is a potential therapeutic target in both conditions Shared vulnerability due to genetic (mtDNA SNPs) and epigenetic (MeCP2) alterations
Neurotrophic Factors (BDNF) downregulation	BDNF and Trk receptors expressed in corneal cells. BDNF expression linked to tear film stability and secretion BDNF receptor gene polymorphism (Val66Met) associated with DED symptoms and severity Trk receptor agonists (small molecules) reduce DED signs via NF-κB pathway modulation	BDNF—a key neurotrophic factor in synaptic function and neural plasticity Reduced peripheral and central BDNF levels in depression, PTSD and anxiety; inversely correlated with symptom severity Val66Met polymorphism linked to increased vulnerability to depression and stress-related disorders Therapeutic strategies enhancing BDNF/Trk signaling under investigation across neuropsychiatric conditions	BDNF/Trk pathway dysregulation common to ocular and mood-related inflammation Val66Met variant impacts both corneal and neural BDNF-mediated function Trk receptor agonists modulate NF-κB signaling → reduced inflammation in both tissues
Serotonin transmission and modulation	Serotonin enhances noradrenaline release from sympathetic nerve endings in the lacrimal gland 5-HT receptors are highly expressed in ocular tissues Activation of 5-HT1A receptors induces oxidative stress, inflammation, and corneal cell damage	Regulates behaviour, mood, and cognitive functions Impacts synaptic function and plasticity Inconsistent findings on the association between depression and reduced serotonin levels	5-HT signaling involved in both mood regulation and ocular surface inflammation Oxidative stress and neuroinflammation as overlapping mechanisms in DED and psychiatric disorders - Potential link between serotonin dysregulation and both emotional and ocular discomfort
Microglial Activation and Neuroinflammation	- Implied through elevated local proinflammatory cytokines - Associated with corneal nerve loss and chronic ocular pain	- Activated microglia release IL-1β and TNF-α - Involved in depressive behaviour, anhedonia, and anxiety-like states	- Peripheral cytokines cross the blood–brain barrier (BBB) and induce microglial priming - Shared neuroimmune mechanisms contribute to central sensitisation in DED and psychiatric disorders

DED: Dry Eye Disease; HPA: Hypothalamic–pituitary–adrenal; PTSD: Posttraumatic Stress Disorder; BDNF: Brain-derived Neurotrophic Factor; SSRIs: Selective Serotonin Reuptake Inhibitors; SNRIs: Selective Serotonin and Norepinephrine Reuptake Inhibitors; CRH: Corticotropin Releasing Hormone; 5-HT: 5-hydroxytryptamine; NE: Norepinephrine; DA: Dopamine; IL: interleukin; TNF: Tumour Necrosis Factor; TGF: Transforming Growth Factor; CRP: C-reactive protein; UV: ultraviolet; MDA: Malondialdehyde; 4-HNE: 4-Hydroxynonenal; ATP: Adenosine Triphosphate; SNPs: Single Nucleotide Polymorphisms; DNA: deoxyribonucleic acid; MeCP2: methyl-CpG-binding protein 2; Trk: tyrosine kinase; RNS: Reactive Nitrogen Species; BBB: Blood–brain barrier; ↑: increased; ↓: decreased; ⟶: leads to.

**Table 2:** Ocular Surface Effects and Management of Common Psychotropic Medications.

**Drug Class/** **Medications**	**Mechanism of Ocular Impact**	**Ocular Symptoms**	**Suggested Management**
SSRIs (Fluoxetine, Sertraline, Escitalopram)	↑ Tear-film serotonin and inflammation Minor anticholinergic effect Tear film instability	Dryness Photophobia Foreign body sensation Blurred vision	Preservative-free artificial tears Consider cyclosporine A Monitor for angle-closure risk Liaison with a psychiatrist
SNRIs (Venlafaxine, Duloxetine)	Altered autonomic tone Meibomian gland dysfunction Rare: IOP elevation, optic neuritis, nystagmus	Burning Blurred vision Photophobia elevated IOP in rare cases	Warm compresses Omega-3 supplements baseline IOP and anterior chamber depth evaluation Adjust dose if ocular effects occur
TCAs (Amitriptyline, Nortriptyline)	Strong anticholinergic activity ↓ Lacrimal and goblet cell secretion Pupil dilation ⟶ blurred vision, and angle-closure risk	Ocular pain Dryness Blurred vision Photophobia Mydriasis, Acute angle-closure risk Cataract	Prefer alternatives with less anticholinergic effect Avoid in elderly and narrow-angle patients Ophthalmic assessment before initiation
Atypical Antipsychotics (Olanzapine, Risperidone, Quetiapine)	Disruption of lipid layer/Meibomian gland function Anticholinergic effects of clozapine Potential epithelial changes or ocular pigmentation at higher cumulative doses	Redness Irritation Visual fatigue Dryness Rarely cataracts or epithelial deposits	Routine ophthalmic evaluations tear film assessment using Schirmer and TBUT Manage blepharitis • Use lubricants or serum drops Consider cyclosporine or serum drops if needed
Benzodiazepines (Diazepam, Lorazepam, Alprazolam)	CNS depression Reduced blink frequency leading to ↓ TBUT and ocular surface dryness Pupil dilation with angle-closure risk	Dryness Incomplete blinking Blurred vision Potential acute angle-closure Diplopia	Blinking exercises • Limit long-term use Regular dry eye screening Ophthalmic assessment before high-dose or long-duration therapy
Mood Stabilizers (Lithium, Valproate)	Altered tear composition Mitochondrial toxicity Ocular motility disturbances Nystagmus	Irritation Burning Visual disturbances (nystagmus, diplopia) Tear film instability	Baseline tear film evaluation (Schirmer, TBUT) Monitor for ocular motility/diplopia Lubricants, if needed

SSRIs: Selective Serotonin Reuptake Inhibitors; SNRIs: Selective Serotonin and Norepinephrine Reuptake Inhibitors; TCAs: Tricyclic Antidepressants; IOP: intraocular pressure; TBUT: Tear Break-up Time test; CNS: Central Nervous System; ↑: increased; ↓: decreased; ⟶: leads to.

**Table 3:** Strategic Research Priorities to Bridge Dry Eye Disease and Psychiatric Disorders.

**Research Focus**	**Objective**	**Recommended Methodology**	**Expected Impact**
Tear-Based Biomarkers	Identify biomarkers that indicate neuroimmune status and psychiatric comorbidity Improve diagnostic precision and disease stratification	Tear proteomics and multiplex cytokine profiling Correlation with validated psychiatric scales (e.g., PHQ-9, GAD-7) Longitudinal biomarker tracking	Enable early diagnosis, patient stratification, and personalised therapy Guide monitoring of disease progression and treatment response
Longitudinal Cohort Studies	Establish directionality and temporality of DED–psychiatric interactions Identify at-risk phenotypes and disease trajectories	Prospective, multi-year observational studies Use of standardised ocular and psychiatric diagnostics Integration of lifestyle, sociodemographic and epigenetic data	Clarify causal pathways and risk factors Support preventive strategies Improve early intervention outcomes
Central Pain and Emotion Circuit Neuroimaging	Assess central sensitisation and dysfunction in affective processing networks in DED Validate neuroimaging biomarkers of neuropathic ocular pain	Resting-state fMRI and diffusion tensor imaging (DTI) with correlation to ocular clinical signs and pain metrics; Brain–ocular functional connectivity analyses Inclusion of both control subjects and distinct DED subgroups	Validate central biomarkers of neuropathic ocular pain Define neurobiological subtypes of DED Guide the development of novel therapies targeting central mechanisms
Gut–Eye–Brain Axis	Investigate the role of gut microbiota on systemic inflammation, neuroimmune function, and ocular–psychiatric outcomes.	Metagenomic and metabolomic profiling Correlate microbial signatures with tear cytokines, cortisol, and BDNF	Identify systemic therapeutic targets Expand the understanding of DED as a systemic disorder
AI and Digital Phenotyping	Develop predictive models for DED–psychiatric comorbidity risk Detect behavioural and emotional patterns associated with DED	Machine learning on integrated datasets (clinical, molecular, behavioural) Smartphone-based digital phenotyping Natural language processing of patient-reported outcomes	Enable precision diagnostics, symptom prediction, and personalised treatment Support remote monitoring and early detection
Integrated Multidisciplinary Care Models	Bridge gaps between ophthalmology, psychiatry, and primary care Improve management of complex comorbid cases	Randomised controlled trials of integrated interventions Shared electronic health records and care pathways Inclusion of patient-reported outcome measures (PROMs)	Enhance clinical outcomes, treatment adherence and patient satisfaction Improve quality of life Reduce healthcare burden

PHQ-9: Patient Health Questionnaire-9; GAD-7: Generalised Anxiety Disorder-7; DED: Dry Eye Disease; fMRI: functional magnetic resonance imaging; DTI: diffusion tensor imaging; BDNF: Brain-derived Neurotrophic Factor; AI: Artificial Intelligence; PROMs: patient-reported outcome measures.

## Data Availability

No new data were created or analyzed in this study. Data sharing is not applicable to this article.
